# Towards universal health coverage for reproductive health services in Ethiopia: two policy recommendations

**DOI:** 10.1186/s12939-015-0218-3

**Published:** 2015-09-30

**Authors:** Kristine Husøy Onarheim, Mieraf Taddesse, Ole Frithjof Norheim, Muna Abdullah, Ingrid Miljeteig

**Affiliations:** Department of Global Public Health and Primary Care, University of Bergen, Postboks 7804, N-5018 Bergen, Norway; Department of Global Health and Population, Harvard T.H. Chan School of Public Health, Boston, USA; United Nations Population Fund, Country Office in Ethiopia, Addis Ababa, Ethiopia

**Keywords:** Reproductive health, Universal health coverage, Inequity, Concentration index, Ethiopia

## Abstract

Reproductive health services are crucial for maternal and child health, but universal health coverage is still not within reach in most societies. Ethiopia’s goal of universal health coverage promises access to all necessary services for everyone while providing protection against financial risk. When moving towards universal health coverage, health plans and policies require contextualized knowledge about baseline indicators and their distributions. To understand more about the factors that explain coverage, we study the relationship between socioeconomic and geographic factors and the use of reproductive health services in Ethiopia, and further explore inequalities in reproductive health coverage. Based on these findings, we discuss the normative implications of these findings for health policy. Using population-level data from the Ethiopian Demographic and Health Survey (2011) in a multivariate logistic model, we find that family planning and use of antenatal care are associated with higher wealth, higher education and being employed. Skilled attendance at birth is associated with higher wealth, higher education, and urban location. There is large variation between Addis Ababa (the capital) and other administrative regions. Concentration indices show substantial inequalities in the use of reproductive health services. Decomposition of the concentration indices indicates that difference in wealth is the most important explanatory factor for inequality in reproductive health coverage, but other factors, such as urban setting and previous health care use, are also associated with inequalities. When aiming for universal health coverage, this study shows that different socioeconomic factors as well as health-sector factors should be addressed. Our study re-confirms the importance of a broader approach to reproductive health, and in particular the importance of inequality in wealth and geography. Poor, non-educated, non-employed women in rural areas are multidimensionally worse off. The needs of these women should be addressed through elimination of out-of-pocket costs and revision of the formula for resource allocation between regions as Ethiopia moves towards universal health coverage.

**Electronic supplementary material:**

The online version of this article (doi:10.1186/s12939-015-0218-3) contains supplementary material, which is available to authorized users.

## Introduction

Although ethical, economic and democratic arguments highlight the importance of health and health investment, not everyone has access to the health services they need [1–3]. Universal health coverage (UHC) has recently been identified as crucial when seeking to improve health and strengthen health systems worldwide. The World Health Organization (WHO) member states endorsed UHC in 2005, a call which gained further support in the World Health Reports in 2010 and 2013. The defined goal of UHC is “to ensure that all people obtain the health services they need without suffering financial hardship when paying for them” [4, 5]. Given resource constraints, this does not entail all possible services, but a comprehensive range of key services that is well aligned with other social goals [6].

A range of socioeconomic, geographic, and cultural factors influence health coverage, but which factors that contribute most differ between settings [7, 8]. Over the last ten to 15 years there has been a call for contextualized empirical quantification of inequalities and factors that contribute to these. This information is necessary when making value judgements about whether the inequalities are unjust inequities, and relevant in academic and policy discussions about provision of health services and non-health services [5, 9–11]. Norheim and Asada suggest that “health inequalities that are amenable to positive human interventions are unacceptable” [12].

Ethiopia is a country with a very unequal distribution of health services [1]. Ethiopia is a low-income country in rapid transition, with high economic growth, positive improvement in development parameters, and impressive reductions in child mortality [13, 14]. According to the recent health sector plans, Ethiopia aims to progressively realise UHC and ultimately to achieve UHC for all Ethiopians [15]. Examples from Afghanistan, Mexico, Rwanda, and Thailand indicate that the goal of achieving UHC can assist in increasing coverage and accelerate equitable progress towards improving women's health [16]. Improving women’s and children’s health is a national priority in Ethiopia [17]. We chose to study reproductive health coverage, which is essential for women’s and children’s health today, and for the health and development of future generations [18].

### Reproductive health in Ethiopia

The Ethiopian Demographic and Health Surveys of 2000, 2005, and 2011 showed that reproductive health coverage in general is very low in Ethiopia, but increasing [19–23]. Descriptive statistics show differences in reproductive health coverage across different strata [19–21], as seen in Table 1.

**Table 1:** Coverage of reproductive health services

	Family Planning^a^		Antenatal care^b^		Skilled attendance at birth^c^	
	Number of observations	Coverage %	Number of observations	Coverage %	Number of observations	Coverage %
Wealth						
Least-poor	2190	48	1644	56	2172	55
Less-poor	1816	27	1227	22	1869	9
Middle	18613	19	1239	15	1863	4
Poorer	2022	17	1351	11	2111	4
Poorest	3478	7	2276	8	3620	3
Location						
Urban	1907	46	1496	56	1985	59
Rural	9612	17	6241	14	9646	5
Education						
No education	7788	15	5167	13	8124	6
Education	3431	36	2570	40	3507	32
Head of Household						
Female headed household	2122	16	1557	25	2183	21
Male headed household	9097	23	6180	21	9448	13
Employment status						
Not employed	7825	18	5296	19	8134	12
Employed	3383	29	2431	30	3480	20
Health insurance						
No health insurance	11155	22	7679	22	11559	14
Health insurance	58	53	50	72	59	73
Age						
15–19 years	514	18	416	17	514	14
20–24 years	2344	26	1594	24	2338	18
25–29 years	3506	22	2283	24	3632	17
30–34 years	2266	21	1501	22	2366	13
35–39 years	1692	21	1195	22	1788	10
>40 years	954	16	748	15	993	7
Birth order						
First birth	2248	29	1471	35	2298	29
Second birth	1963	28	1331	30	2022	20
Third birth	1630	21	1078	19	1686	12
Fourth birth	1408	18	970	18	1458	10
Fifth or subsquent birth	3970	16	2287	14	4167	6
*Reporting problem*						
Permission to go						
Problem	3784	15	2477	12	3927	7
Not a problem	7433	25	5254	27	7695	18
Getting money						
Problem	7826	18	5283	17	8095	10
Not a problem	3392	28	2449	32	3528	24
Distance to facility						
Problem	8304	17	5552	15	8594	8
Not a problem	2912	35	2178	40	3027	32
Transportation						
Problem	8697	18	5824	16	9002	9
Not a problem	2520	35	1907	41	2620	33
Going alone						
Problem	7014	19	4733	18	7273	10
Not a problem	4202	26	2998	29	4348	22
No female provider						
Problem	7178	18	4800	17	7435	10
Not a problem	4037	28	2931	30	4185	21
No provider						
Problem	7557	19	5087	19	7821	11
Not a problem	3661	28	2645	29	3802	20
No drugs						
Problem	7753	19	5237	19	8031	11
Not a problem	3465	28	2495	29	3592	21
Workload at home						
Problem	7511	19	5030	17	7782	10
Not a problem	3701	27	2698	31	3835	23
Religion						
Muslim	5211	14	3350	17	5435	11
Protestant	2180	22	1476	18	2233	10
Orthodox	3485	34	2680	31	3613	22
Other religion	338	11	227	11	345	6
Region						
Tigray	1164	21	846	30	1202	11
Affar	1105	5	713	8	1128	5
Amhara	1226	30	959	12	1291	9
Oromiya	1694	23	1100	19	1759	9
Somali	953	3	559	8	1027	8
Benishangul-Gumuz	982	20	670	15	1015	8
SNNPR	1576	23	1051	17	1612	6
Gambela	834	18	605	23	847	17
Harari	626	31	439	34	659	32
Addis Ababa	383	68	344	87	399	85
Dire Dawa	676	22	451	36	692	35
Total	11219	22	7737	22	11631	14

^a^Family planning; women who said they did not want more children or that they would like to wait two more years before they have another child, and who are not currently pregnant

^b^Antenatal Care: ≥ four antenatal visits during pregnancy

^c^Skilled Birth Attendance: birth assistance by a doctor, nurse or midwife, health extension worker or other health professional among women who gave birth the last 5 years

Source: Central Statistical Agency & ICF International. 2012. Ethiopia Demographic and Health Survey, 2011. Addis Ababa, Ethiopia: Central Statistical Agency and ICF International

**Fig.1:**
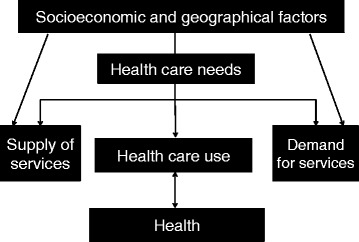
Factors impacting reproductive health and health coverage

In 2008, the Ethiopian Federal Ministry of Health and collaborating partners carried out a national baseline assessment of the availability, use and quality of emergency obstetric and newborn care services, in order to better understand the delivery of care to Ethiopian women giving birth [24, 25]. Few facilities provided care according the recommended WHO standards and only 7 % of all deliveries occurred in institutions, one of the lowest proportions in the world. Both “push and pull factors” impact whether and when women make use of delivery-care services; these include sociocultural factors, economic accessibility, perceived benefit from and need of services, and physical accessibility [26]. These can be understood as supply and demand factors, as illustrated in Fig. 1.

Although health equity is a stated goal in the Ethiopian policy plans, an equity lens has only been applied up to a certain level in health research relevant to policymaking. Policymakers face dilemmas such as whether to target certain groups in need of particular services in a population, or to promote universal care for the whole population. The World Health Organization Consultative Group on Equity and Universal Health Coverage suggested a three-part strategy to secure a progressive realization of UHC and equity on the path to UHC:

1. Categorise services into priority classes.
2. Increase coverage for high-priority services to everyone and reduce out-of-pocket payments.
3. Ensure that disadvantaged groups are not left behind [6].

To make fair choices on the path to UHC in Ethiopia, the recommendations from the WHO expert group presuppose contextualised empirical data on reproductive health and systematic analysis of how different explanatory variables relate to reproductive health coverage and inequalities in health coverage [23]. Knowledge of the current situation is the basis for a proper ethical analysis that could guide policy making and planning. As noted by Norheim and Asada, definitions and measures of inequity in health should be better integrated with theories of distributive justice [12].

### Purpose of study

In this paper, we attempt to fill in some of the knowledge gap about reproductive health coverage indicators in Ethiopia and link it to a normative discussion of distributive justice and health. In the first part of this paper we aim to identify possible associations between socioeconomic and geographic factors and coverage of met need for family planning, use of antenatal care, and skilled attendance at birth. Using concentration indices, we quantify inequalities in coverage and look at how identified socioeconomic and geographic factors are associated with these inequalities by decomposition of the concentration indices. In the second part of this paper we discuss the normative implications of these findings for health policy in Ethiopia.

## Methods

### Measures of inequality in reproductive health

#### Data material

Survey data have the greatest potential in the analysis of health equity [27]. We used data from the most recent Ethiopian Demographic and Health Survey (EDHS 2011), conducted by the Ethiopian Central Statistical Agency between December 2010 and June 2011 [21]. This household-level survey is a nationally representative sample of 17,817 households selected on the basis of the Population and Housing Census from 2007 (Ethiopian Central Statistical Agency). The sample was selected by a stratified cluster sampling design and consisted of 16,515 women (15–49 years of age) and 14,100 men (15–59 years of age). Data design and collection is fully described in the Ethiopia Demographic and Health Survey 2011 final report [21].

### Ethical approval

Ethical clearance for the EDHS was provided by the Ethiopian Health and Nutrition Research Institute Review Board, the National Research Ethics Review Committee at the Ethiopian Ministry of Science and Technology, the Institutional Review Board of ICF International, and the U.S. Centers for Disease Control and Prevention. The current study was exempted from full review by the Regional Committee for Medical and Health Research Ethics in West Norway, as the study is based on anonymous data with no identifiable information.

### Variables of interest

As the overall reproductive health coverage is low in Ethiopia [21], we studied individual-level indicators proposed by the WHO to monitor reproductive health [28]. The following indicators for reproductive health coverage have been identified as high-priority interventions in the Ethiopian Health Sector and Development Plan IV [17]: family planning (FP), antenatal care (ANC), and skilled birth attendance (SBA) (see web-Additional file 1).

In the analysis we explanatory variables were based upon descriptive data (Table 1) and recommendations from the current literature on factors that have been associated with reproductive health coverage and mortality, and factors that have been recognised as relevant in inequality analysis [26, 29, 30]. We included a range of possible explanatory variables that have been shown to be associated with reproductive health services: socioeconomic variables at the household level, barriers reported at the household level, geography, and use of other health care services. Maternal age and birth order of child were included in the analysis as potential confounding factors [23].

We used the wealth index from the EDHS as a proxy for socioeconomic status. The index was created using principal component analysis, where the index is a continuous variable based on household assets and living standard (for further details, see the DHS website [31]). Based on the wealth index, five wealth quintiles were used in the multivariate analysis, as our primary interest was the difference between poor and less-poor groups.

We included additional socioeconomic factors as dummy variables (for further description, see the web-Additional file 1).

To further understand the barriers to health-service use [26], we included reported problem(s) of getting medical help for self in the model. Although we cannot assume a causal relationship between the reported problem(s) of “getting medical help for self” and health coverage; studying the reported problems can add information about less understood household level barriers and demand factors (Fig. 1) [26]. We included the following reported problems in our analysis (0 = not a problem, 1 = a significant problem): permission to go, money needed for treatment, distance to health facility, having to take transportation, not wanting to go alone, concern over no female provider, concern over no provider, concern over no drugs being available, and workload inside and outside the home. These factors may explain reproductive health coverage and inequalities in reproductive health coverage.

To determine if identified religious beliefs and related traditions were associated with health coverage, we included information related to religious view (Islam, Orthodox Christianity, Protestant Christianity, and other religions). We also included administrative region (nine regions and two cities) as independent variables to determine if they would be associated with coverage. We used Addis Ababa as a reference region, as this is the region that is closest to reaching full coverage of services (Table 1).

Previous use of antenatal care and skilled attendance at birth were included in the models, as the literature indicates that previous health-services utilisation is a predictor for successive use of health services (see web-Additional file 1) [23]. The analysis was conducted using STATA statistical software (STATA 13.1).

### Regression analysis

To explore possible associations between explanatory variables and binary outcomes, other factors held equal, we performed multivariate logistic regression [32]. The data material is from a household survey, and standard sample weights (provided in the DHS data set) were used to correct for potential over-and under-sampling. Further, we adjusted for the clusters (the primary sampling units). The analysis was based on women in their reproductive age (15–49 years); 11,654 women, and their 7764 last pregnancies. As previous health care use and use of antenatal care was included in the model, the analysis was limited to 7422; 7708; and 7702 women in the final regression analysis of family planning, antenatal care and skilled attendance at birth, respectively.

Modifying the outcome of the logit model, we present the exponential coefficients as adjusted odds ratios (OR) to give the reader an approximation of how a 1-unit change in the explanatory variables will affect the dependent variable(s); If the OR is higher than one, exposure associated with higher odds of the outcome. If the OR is lower than one, exposure is associated with lower odds of the outcome.

Based on the current literature and Table 1, we hypothesised that higher education, higher wealth, urban residence, being employed, and having health insurance would be associated with higher use of reproductive health services [19–21, 26, 29, 33, 34]. We further hypothesised that female headed household and problems reported with getting medical help for self would be factors associated with a lower chance of using reproductive services.

It is difficult to predict how religion and geography affect outcomes, but the descriptive data indicate that they may have an impact (Table 1).

### Inequality analysis

The concentration index has been used to quantify health and health service coverage inequalities when seeking to understand how coverage indicators of interest vary across income or wealth distributions [27]. Recent discussions illustrate that none of the inequality measures available are perfect [35]. We chose the Erreygers corrected concentration index (CCI), as it corrects for several problems in the standard concentration index as noted in the literature [7, 35]. For the reproductive health coverage variables of interest (*y*), the Erreygers CCI can be calculated as:

1 $$ CCI(y)=8\operatorname{cov}\left({y}_i{R}_i\right) $$

where *y*_*i*_ is reproductive health coverage (dependent variable) of the individual *i* and *R*_*i*_ is her fractional rank in the wealth distribution, with *i* = 1 for the poorest individual and *i* = N for the least-poor individual in the sample.

A positive CCI will indicate that the better off have disproportionately higher service coverage, and the opposite is true for a negative CCI. We hypothesise that the CCI will be positive when looking at FP, ANC, and SBA, as the literature has described that the better off make more use of services [1, 7, 36–38]).

To further explore which factors contribute to inequalities, the concentration index can be decomposed by relating health outcomes to their potential socioeconomic determinants [27, 35, 39]. Hereby, we can investigate underlying inequalities that may explain the variation in health coverage. The concentration index can be decomposed to the contributions of the individual factors to wealth-related health inequality, where each factor’s contribution is the product of its sensitivity and the degree of wealth-related inequality of the given factors [27, 35, 39]. The concentration index of a given dependent variable of interest, *y*, can be written as

2 $$ CCI(y)=4\left\{{\displaystyle {\sum}_k\left({\beta}_k{\overset{-}{x}}_k\right)}C{I}_k+G{C}_{\varepsilon}\right\} $$

where ${\overset{-}{x}}_k$ is the mean of *x*_*k*_ (reproductive health coverage), *CI*_*k*_ is the CI of x_k_, and *GC*_*ϵ*_ is the generalised CI of the error term (ε). *CCI* is then equal to a weighted sum of the CIs of the *k* regressors. The residual expresses the inequality that cannot be explained due to systematic variation in the regressors included in the analysis. The closer the residual goes towards 0, the better the fit of the model. We use the wealth index as a continuous variable creating the fractional rank, but look at the contribution of the different wealth quintiles in the decomposition analysis.

The decomposition of the dependent variable is based on a linear regression model. Though logistic regression was used in the multivariate analysis, Gravelle et al. have shown that the decomposition analysis can also be extended for binary outcomes [40]. Only explanatory factors that showed *P* < 0.05 significance in the multivariate regression analysis were included in the decomposition analysis.

## Results

### Determinants of reproductive health coverage

Socioeconomic and geographic factors associated with reproductive health coverage are shown in Table 2 (only significant results are shown, *P* < 0.05).

**Table 2:** Multivariate logistic regression analysis. Odds Ratio

	Family Planning	Antenatal Care	Skilled Birth Attendance
Wealth			
Poorest	0.270^***^	0.301^***^	0.237^***^
Poorer	0.436^***^	0.419^***^	0.336^***^
Middle	0.452^***^	0.485^***^	0.294^***^
Less-poor	0.653^*^	0.674^*^	0.492^***^
*Least-poor*	1.000	1.000	1.000
Education	1.347^**^	1.865^***^	2.144^***^
Urban	0.939	1.159	3.357^***^
Female headed household	0.484^***^	0.940	1.326
Employed	1.581^***^	1.449^***^	1.299
Birth order			
Second birth	1.415^*^	0.905	0.508^***^
Third birth	1.324	0.612^*^	0.553^*^
Forth birth	0.968	0.694	0.309^***^
Fifth or subsequent birth	0.869	0.664^*^	0.323^***^
*First birth*	1.000	1.000	1.000
Reported problem			
Getting permission to go	1.084	0.697^**^	0.808
Religion			
Protestant	1.724^**^	0.714	1.343
Orthodox	1.676^**^	1.091	1.937^***^
Other religion	0.733	0.678	1.151
*Muslim*	1.000	1.000	1.000
Region			
Affar	0.383^**^	0.079^***^	0.288^***^
Amhara	1.091	0.069^***^	0.417^**^
Somali	0.129^***^	0.044^***^	0.597
Benishangul-Gumuz	0.793	0.122^***^	0.657
SNNPR	0.719	0.145^***^	0.367^*^
Gambela	0.748	0.263^***^	1.267
Harari	0.739	0.152^***^	1.250
Dire Dawa	0.567^*^	0.212^***^	2.565^**^
Oromiya	0.752	0.129^***^	0.503^*^
Tigray	0.486^**^	0.193^***^	0.254^***^
*Addis Ababa*	1.000	1.000	1.000
Previous health care use			
Antenatal care	1.904^***^		3.012^***^
Skilled attendance at birth	1.564^**^		
*N*	7422	7708	7702
pseudo *R* ^2^	0.138	0.175	0.403

Exponentiated coefficients

^*^ *p* < 0.05, ^**^ *p* < 0.01, ^***^ *p* < 0.001

### Family planning

Lower wealth, female headed household, and living in the administrative regions Affar, Somali, and Tigray are associated with lower coverage (*P* < 0.05). In our model, education, being employed, being Protestant or Orthodox, and previous use of ANC and SBA is associated with higher coverage of family planning (*P* < 0.05).

### Antenatal care

Lower wealth, reported problem with getting permission to go, and all administrative regions (compared to Addis Ababa) are associated with lower ANC coverage (*P* < 0.05). Use of ANC is associated with higher education and being employed (*P* < 0.05).

### Skilled birth attendance

Higher SBA is associated with education, urban location, being orthodox, living in Dire Dawa, and previous use of ANC (*P* < 0.05). Lower wealth, later birth order, and the administrative regions of Affar, Amhara and Tigray are associated with lower SBA coverage.

Age and self-reported problems, with the exception of permission to go related to ANC, did not show significant associations with coverage.

Inequalities in reproductive health coverage

Table 3 shows degree of inequality in use of reproductive health coverage, measured by the Erreygers concentration index. FP, ANC, and SBA show pro-rich distributions with CCIs of 0.274, 0.278 and 0.263, respectively.

**Table 3:** Erreygers Corrected Concentration Indices

Family planning	Antenatal Care	Skilled Birth Attendance
0,274	0,278	0,263

The decomposition of the CCIs shows contributions to inequalities in reproductive health coverage based on associations to the outcomes of interest and/or the factors’ unequal wealth distribution (concentration index) (Table 4). Wealth, when summarised across contributions from the different wealth quintiles, is the most important contributor to inequality: 59 % for family planning, 58 % for ANC, and 32 % for SBA. Previous ANC and SBA explain 13 % and 10 % of the inequality in FP. Living in Addis Ababa contributes to 10 % of the inequality in ANC use. Urban location, previous ANC, and education explain 38 %, 13 %, and 11 %, respectively, of the inequality in SBA.

**Table 4:** Decomposition of Erreygers Corrected Concentration Indices

	Unmet Need for Family Planning		Antenatal Care		Skilled Birth Attendance	
	Absolute Contribution	% contribution	Absolute Contribution	% contribution	Absolute Contribution	% contribution
Wealth						
Poorest	0,000	0,0	0,175	62,9	0,000	0,0
Poorer	−0,018	−6,7	0,064	22,9	−0,002	−0,6
Middle	0,006	2,2	−0,019	−6,7	0,000	−0,2
Less-poor	0,055	20,2	−0,059	−21,0	0,004	1,5
Least-poor	0,119	43,4	0,000	0,0	0,081	30,9
Education	0,022	8,1	0,038	13,7	0,028	10,7
Urban	-	-	-	-	0,099	37,5
Female headed household	−0,003	−1,1	-	-	-	-
Employed	0,011	4,1	0,007	2,6	0,002	0,7
Religion						
Protestant	0,000	0,0	-	-	0,000	0,0
Orthodox	0,000	0,1	-	-	0,004	1,3
Other religion	0,004	1,3	-	-	0,000	0,1
Muslim	0,004	1,6	-	-	0,001	0,3
Region						
Affar	0,000	0,0	0,001	0,5	0,003	1,1
Amhara	−0,005	−1,7	0,004	1,5	0,009	3,4
Somali	0,001	0,5	0,003	1,1	0,004	1,6
Benishangul-gumuz	0,000	−0,1	0,000	0,1	0,001	0,4
SNNRP	−0,001	−0,4	0,003	1,0	0,008	3,1
Gambela	0,000	0,0	0,000	0,0	0,000	0,1
Harari	0,000	0,1	0,000	0,0	0,000	−0,2
Dire Dawa	0,000	0,0	0,000	0,1	0,000	0,0
Oromiya	0,003	0,9	−0,004	−1,3	−0,011	−4,2
Tigray	0,001	0,2	−0,001	−0,3	0,003	1,3
Addis Ababa	0,009	3,3	0,028	10,0	0,002	0,9
Previous health care use						
Antenatal Care	0,036	13,1	-	-	0,035	13,4
Skilled Birth Attendance	0,027	9,7	-	-	-	-
Residual	0,003	1,2	0,036	13,1	−0,008	−3,1
Total	0,274	100,0	0,278	100,0	0,263	100

Explanatory variables included based on the logistic multivariate regression (*p* < 0.05)

## Discussion

Towards universal health coverage for reproductive health services in Ethiopia: Still a long way to go

Coverage for reproductive health services is very low in Ethiopia. The majority of Ethiopian women do not make use of essential reproductive health care services. Coverage for family planning is 22 %; for antenatal care 22 %, and for skilled birth attendance 14 %. As noted in the WHO report “Making fair choices on the path to universal health coverage”, this coverage gap is the greatest unfairness [6]. The maternal mortality rate in Ethiopia is among the highest in the world [41], and further reductions cannot be expected until coverage is substantially increased – and quality of services improved [24].

In addition, our analysis shows that several socioeconomic and geographic factors are associated with inequalities in reproductive health coverage. Wealth, education, employment, and urban location are of particular importance for higher coverage. There is substantial regional variation in coverage when compared to Addis Ababa (the capital); in particular, Affar lags behind. Gwatkin and Ergo have pointed out that policymakers can choose between scaling up interventions for all people or targeting the worse off or the poor through “progressive universalism” [42]. They argued for progressive universalism when moving towards UHC, an idea that has been supported by the recent Lancet Commission on Investing in Health [3]. Based on our analysis, women who are poor, have little education, live in rural locations, and are not employed should be targeted if this progressive approach is chosen.

Our study finds high inequality across the reproductive health coverage indicators. These findings highlights that average coverage levels might hide an uneven distribution of services within populations. Bonfrer et al., also using the Erreygers CCI, report similar, but slightly lower CCI values when looking at antenatal care and skilled attendance at birth in Ethiopia [7]. However, our finding that inequality (measured as CCI) is almost as high among the three indicators of interest (FP (CCI = 0.274), ANC (CCI = 0.278), and SBA (CCI = 0.263) is new, as the previous literature finds that inequality in SBA and other treatment interventions is especially high [1, 43, 44].

Reproductive health services are defined as essential – and high priority – services in Ethiopia. This means that family planning, antenatal care, and skilled birth attendance should be accessible and used by all who need them. Although maternity services are formally provided for free in Ethiopia, Pearson et al. showed that 65 % of hospitals and health centres charge for maternal care [45]. According to the national health account from 2014, household covered 28 % of the total reproductive health spending. Though national health expenditure per capita increased from US$16 to US$21 between 2007/08 and 2010/11, this is far below the recommended minimum of US$44 per capita by WHO [46]. For those facing financial hardship, user fees, transport costs, and other supply-side factors are likely to make the choice to obtain necessary health services more difficult. WHO’s Consultative Group on Equity and UHC recommends that patient costs should be eliminated for high priority services. This is justified both in terms of efficiency and equity [6].

### Salient findings and policy recommendations

#### Wealth is the most important factor for inequality: All patient costs should be eliminated

The decomposition analysis enables us to study contributions to inequality in coverage in greater depth. Using findings from the multivariate regression analysis, where we study associations between explanatory factors and average coverage, our decomposition analysis shows that difference in wealth is the major contributor to inequality in health coverage. McKinnon et al. decomposed inequality in cervical cancer screening rates, and found large heterogeneity in the impact of different contributors to inequality in screening rates in 67 countries [8]. This finding emphasises the importance of a contextualised inequality analysis. The major contributors to inequality in our analysis are closely related to the most important determinants of coverage in the regression analysis. Even though several factors are significantly associated with reproductive health coverage, and there is some variation in the magnitude of the different factors, wealth is clearly the most important factor for the inequality.

Depending on whether the aim is to improve service coverage alone, or to reduce inequality in coverage, the appropriate policy might differ. The most important aim should be to increase coverage for all. Addressing all factors determining supply and demand is therefore warranted. Second, to reduce unfair inequalities in reproductive health coverage, inequality in wealth is the most important contributor and should be addressed through eliminating all patient costs. Wealth is also found to be associated with average health coverage, but its importance to inequality in coverage is not captured in the multivariate regression analysis. Inclusion of a concentration index analysis is therefore key to understanding the factors contributing to inequality in health coverage.

#### Regional and geographic inequality: The formula for resource allocation between regions should be revised

We found significant regional differences, and this may indicate that there are structural or cultural differences within Ethiopia that affect reproductive health coverage. The Annual Performance Report on the Ethiopian Health Sector and Development Plan from 2012 to 2013 has shown that allocated financing for health services differs between the administrative regions, with regional budgets allocated to the health sector ranging from 6.8 % in Addis Ababa to 14.7 % in Dire Dawa, with a national average of 9.75 % [47]. These geographic inequalities could be reduced by a more fair allocation of resources [6, 48]. Supply-side of services from the public and private sector, and the quality of these services, are known to impact the use of services [24, 49]. The Ethiopian survey of Emergency Obstetric and Neonatal Care found that there were only 83 comprehensive and basic emergency obstetric care facilities in 2007, which was 11 % of the 739 facilities recommended by the WHO. There were large differences between regions, both in terms of number of facilities per population and whether the facilities met signal functions [24]. In particular, the Affar and Somali regions (with predominantly semi-pastoralist populations) were lagging behind. Though scaling up maternal and child health services have been a priority after 2007, revision of the formula for resource allocation between regions should be considered as Ethiopia moves towards universal reproductive health coverage

### Strengths and limitations

We used cross-sectional national population-based survey data from the Ethiopian DHS from 2011. By adjusting for sample weights and clustering, we aimed to correct for differences in probability in our sample. The DHS provides rich health and non-health data and was collected and reported in a systematic manner. The overall response rate of the survey was high (95 % for women, 89 % for men), and the risk of selection bias was relatively low. However, our analysis focused on women who gave birth the 5 years prior to the survey and the utilization of services related to their last pregnancies (7764). We cannot rule out that these women may differ from the women who were not pregnant, which may have impacted the results (see web-Additional file 1). There were missing data on some of the outcome and explanatory variables, which could contribute to potential bias. However, more than 95 % of the women in their reproductive age who had given birth were included in the regression models for FP, ANC and SBA. Some may disagree that health extension workers should be classified as “skilled birth attendants”, but as health extension workers are key components of the national health system in Ethiopia, we chose to include them as skilled attendants [47].

Our analysis of the Ethiopian data provides a contextualised and robust analysis relevant to evidence-informed policymaking and health-and welfare-planning. Our analysis included a broad range of factors to avoid potential confounding of the results. However, we are not able to fully capture more proximal factors that influence health coverage, such as cultural factors and quality of care. Ethiopia is a country with cultural diversity, and the analyses do not fully account for this. The R2 ranges between 0.14 (FP) and 0.40 (SBA). This may indicate that factors other than those included in our model may better explain family planning. As DHS data are household-level data, we do not know whether the observed associations are due to intra-household decision-making (cultural norms, behaviour, out-of-pocket expenses, etc.) or external factors (technical provision of services or goods, etc.) [27]. The included “report of problem” factors illustrate potential barriers that were not found to give significant results. As this is a cross-sectional study, we cannot rule out reverse causality.

By using the Erreygers CCI, we make use of one of the newest and most comprehensive methodologies for analysis of socioeconomic inequality [35]. By including a range of possible explanatory variables from the multivariate regression analysis, we are able to study not only socioeconomic inequality, but also how other factors are associated with the inequality in reproductive health coverage. After completion of our analysis, a supplementary mini-DHS for reproductive health services was published [50]. Although the mini-DHS shows some improvements, we do not believe these data would change our conclusions.

## Conclusion

Ethiopia is starting on the path to universal health coverage, aiming *inter alia* to provide reproductive health services to all. In depth understanding of coverage gaps and inequalities in coverage is crucial for efficient and fair health policies. Our study re-confirms the importance of a broader approach to understanding reproductive health coverage, and in particular the importance of inequality in wealth and geography. Poor, non-educated, non-employed women living in rural areas are multidimensionally worse off in terms of access to reproductive health services, and the needs of these women could be addressed through elimination of all patient costs and revision of the formula for resource allocation between regions as Ethiopia moves towards universal reproductive health coverage.

## Additional file

Additional file 1:
**Description of variables.** (DOC 30 kb)
