# Supplementary material for: Towards universal health coverage for reproductive health services in Ethiopia: two policy recommendations
Source: Int J Equity Health. 2015 Sep 30;14:86. doi: 10.1186/s12939-015-0218-3 (PMC4588686; doi:10.1186/s12939-015-0218-3)
Supplement: Additional file 1: — Description of variables. (DOC 30 kb) [file 12939_2015_218_MOESM1_ESM.docx]

# Web-appendix A: Description of variables

## Dependent variables

a) Family planning (FP), defined as the avoidance of pregnancy through modern contraception by a woman who is not currently pregnant and who preferred to wait a year or more before they had another child, were in need of family planning.

b) Antenatal care (ANC), defined as ≥4 antenatal visits during (the last) pregnancy among women who have given birth in the last five years, as recommended by the WHO.

c) Skilled birth attendance (SBA), defined as birth assistance by a doctor, nurse, midwife, health extension worker or other health professional, among women who have given birth in the last five years (last pregnancy).

Dummy variables were created so that FP, ANC and SBA were coded 0 if not used and 1 if used.

## Independent variables

We included additional socioeconomic factors as dummy variables: education (0 = no education; 1 = primary, secondary or higher education), urban residence (0 = rural, 1 = urban), sex of head of household (0 = male, 1 = female), employed (0 = currently not employed, 1 = currently employed). Although health insurance coverage was low, we included it as a proxy for financial protection (0 = not covered, 1 = covered).

To determine if identified religious beliefs and related traditions were associated with health coverage, we included dummies based on religious view (Islam, Orthodox Christianity, Protestant Christianity and other religions). We constructed binary dummies for the nine regions and two cities (Addis Ababa and Dire Dawa) as independent variables to determine if they would be associated with coverage. We used Addis Ababa as a reference region, as this is the region that is closest to reaching full coverage of services (Table 1).

Previous use of antenatal care (ANC) and skilled attendance at birth (SBA) were included in the models, as the literature indicates that previous health-services utilisation may be a predictor for successive use of health services. Hence, also the FP analysis, we focus on women who were pregnant the last five years. When we run the regression models without including ANC and SBA, the pseudo R2 is lower (results available upon request). The high R2 when including ANC and SBA may be related to a better model (that previous health care use as an important factor to use of family planning), a selection of more similar women in the model (when only looking at those with an earlier pregnancy) or other reasons. As our model build upon previous work indicating that previous health care use may be of importance, we chose to include ANC and SBA in the model.
